# Reasonable expectations of privacy in non-disclosure of familial genetic risk: What is it reasonable to expect?

**DOI:** 10.1016/j.ejmg.2018.11.013

**Published:** 2019-05

**Authors:** Victoria Chico

**Affiliations:** Society and Ethics Research Group, Connecting Science, Wellcome Genome Campus, Cambridge, UK

**Keywords:** Confidentiality, Genetic information, Non-disclosure, Privacy, Reasonable expectation of privacy

## Abstract

Where there is conflict between a patient's interests in non-disclosure of their genetic information to relatives and the relative's interest in knowing the information because it indicates their genetic risk, clinicians have customarily been able to protect themselves against legal action by maintaining confidence even if, professionally, they did not consider this to be the right thing to do. In *ABC v St Georges Healthcare NHS Trust* ([2017] EWCA Civ 336) the healthcare team recorded their concern about the wisdom of the patient's decision to withhold genetic risk information from his relative, but chose to respect what they considered to be an unwise choice. Even though professional guidance considers that clinicians have the discretion to breach confidence where they believe this to be justified, (Royal College of Physicians, Royal College of Pathologists and the British Society of Human Genetics, 2006; GMC, 2017) clinicians find it difficult to exercise this discretion in line with their convictions against the backdrop of the legal prioritisation of the duty to maintain confidence. Thus, the professional discretion is not being freely exercised because of doubts about the legal protection available in the event of disclosure. The reliance on consent as the legal basis for setting aside the duty of confidence often vetoes sharing information with relatives. This paper argues that an objective approach based on privacy, rather than a subjective consent-based approach, would give greater freedom to clinicians to exercise the discretion which their professional guidance affords.

## 1 Introduction

The position of consent as a lawful basis for setting aside the duty of confidence provides the basis for determining how competing interests are valued in the context of the sharing of familial genetic information. Where the patient consents, the clinician will disclose, or facilitate disclosure. Where the patient does not consent, clinicians often withhold the information from relatives, even if they do not consider this to be the right thing to do (Clarke et al., 2005). This is because of the key position of consent in negating an action in breach of confidence. Some jurisdictions in particular the US are more content to recognise a duty to disclose to relatives at-risk of genetic conditions. However, the position in most of Europe is that disclosure is permitted with the consent of the data subject only (Godard et al., 2006). Despite the domination of consent in setting aside the duty of confidence, in the context of processing personal and confidential health data more generally, there has been a shift away from reliance on consent as the lawful basis for processing. European Regulation provides a number of alternative bases to consent for the processing of personal health data (General Data Protection Regulation) and this move away from consent is also evident in the national policy position (https://www.hra.nhs.uk/hra-guidance-general-data-protection-regulation/file:///C:/Users/lw1vlc/AppData/Local/Temp/igagdprconsent-1.pdf; https://ico.org.uk/for-organisations/guide-to-the-general-data-protection-regulation-gdpr/consent/what-is-valid-consent/; https://mrc.ukri.org/research/facilities-and-resources-for-researchers/regulatory-support-centre/gdpr-resources/; https://assets.publishing.service.gov.uk/government/uploads/system/uploads/attachment_data/file/535024/data-security-review.PDF; https://www.gov.uk/government/uploads/system/uploads/attachment_data/file/668727/830_-_Supporting_health_and_care_professionals_to_share_data_in_line_with_patient_expectations_-_October_2017_seminar_FINAL.pdf) This piece considers how competing familial interests in disclosure might work if consent is not the only lawful basis for setting aside the duty of confidence. Instead, it is argued that the concept of privacy, which is being increasingly recognised as the interest protected by the obligation of confidence, through the influence of Article 8 of the European Convention on Human Rights, could provide a mechanism for valuing legal interests in disclosure and non-disclosure of familial genetic information. This development would enable clinicians to exercise the discretion that professional guidance affords.

This discussion begins with a consideration of the general move away from consent as providing a lawful basis for processing persona data and confidential patient information. Following this, there is an investigation the scope of the interest in confidentiality as a means of protecting decisions not to disclose genetic information which would benefit relatives. Within this discussion, there is an analysis of how professional guidance has historically underpinned the development of legal standards of care. This leads into a consideration of the development of legal principles in the context of breach of confidence outside the medical relationship context. Here the article analyses how the concept of ‘reasonable expectations of privacy’ might provide the basis for determining whether there has been a breach of the duty of confidence. Following this discussion, the piece investigates how the concept of a ‘reasonable expectation of privacy’ might map onto conflicts between maintaining confidence and permitting disclosure that arise in the event of a patient's refusal to share relevant genetic information with at-risk relatives.

## 2 The move away from consent as the lawful basis for processing personal data

The General Data Protection Regulation (GDPR) provides five bases, other than consent, upon which personal data can be lawfully processed. The Regulation recognises the importance of the protection of personal data, but cautions that the right to protection of personal data is not an absolute right and must be interpreted proportionately where the processing of personal data is designed to protect mankind. In line with this move away from consent, the national policy position is that the basis for processing data for health and social care research under the GDPR should not be consent.(https://www.hra.nhs.uk/hra-guidance-general-data-protection-regulation/; file:///C:/Users/lw1vlc/AppData/Local/Temp/igagdprconsent-1.pdf; https://ico.org.uk/for-organisations/guide-to-the-general-data-protection-regulation-gdpr/consent/what-is-valid-consent/; https://mrc.ukri.org/research/facilities-and-resources-for-researchers/regulatory-support-centre/gdpr-resources/). In a similar vein, the National Data Guardian (NDG) has recognised that the principle of implied consent is becoming an increasingly unsuitable legal basis for setting aside the duty of confidence in the context of evolving new models of care (https://assets.publishing.service.gov.uk/government/uploads/system/uploads/attachment_data/file/668727/830__Supporting_health_and_care_professionals_to_share_data_in_line_with_patient_expectations_-_October_2017_seminar_FINAL.pdf).

The NDG is currently consulting on a model that:

departs more radically from current practice by adopting reasonable expectation as an alternative to implied consent. Instead of inferring whether the patient had consented, health and care professionals (and potentially the courts) would ask whether use or disclosure would be a reasonable expectation of a patient in the circumstances (respecting any expression of dissent.) On this basis disclosure would be justified even without the patient's explicit or implicit consent.(https://assets.publishing.service.gov.uk/government/uploads/system/uploads/attachment_data/file/668727/830__Supporting_health_and_care_professionals_to_share_data_in_line_with_patient_expectations_-_October_2017_seminar_FINAL.pdf)

The NDG's work further demonstrates a shift away from individual consent as a basis for determining when sharing confidential patient information will be lawful in favour of a general adoption of a concept of reasonable expectations which seeks to strike a proportionate balance between individual privacy and society's competing interests in sharing information on the basis of what people would reasonably expect.

The importance of this NDG work has been recognised in the context of genetic medicine. In October 2016, the Association for Clinical Genetic Science (ACGS) and the PHG Foundation collaborated in delivery of an evidence session for the National Data Guardian which sought to engage NDG assistance in addressing a challenge within the field of genomic medicine. The challenge relates to inconsistent understanding of the legality of sharing genetic data about one person to aid the interpretation of clinical significance of genetic test results returned to another. Given consent can only be implied in disclosure for purposes relating to an individual's own care. Disclosure of identifiable data to a clinician with no responsibility for an individual's care, in order to inform the care provided to another, falls outside that traditional understanding.(https://assets.publishing.service.gov.uk/government/uploads/system/uploads/attachment_data/file/644689/546_Developing_a_consensus_on_data_sharing_to_support_NHS_clinical_genetics_and_genomics_services_FINAL.pdf).

## 3 The position of professional guidance in providing content for legal standards of care

In *ABC* the daughter of a male patient brought an action against his clinicians for their failure to warn her about her father's Huntington's disease. The health professionals looking after the father had sought his consent to disclose his diagnosis to his daughter, which he had refused. The daughter, who had been pregnant at the time of the non-disclosure, argued that she should have been told of her father's hereditary condition, so she could be aware of her own risk and that of her unborn child. If she had been told, she argues that she would have elected to terminate her pregnancy. Her case was struck out by the High Court on the basis that there was no reasonable cause of action ([2015] EWHC 1394 (QB)). The crux of the case was whether there was a duty of care to third parties, rather than whether the clinicians owed the father a duty of confidence, as they had not breached his confidence. However, given that the clinicians questioned the wisdom of his decision, we can assume that, if the confidentiality point had not arisen, the daughter would have been told of her genetic risk, thereby avoiding this litigation. The Court of Appeal disagreed with the High Court and found that the issue of whether it is just, fair and reasonable to impose a duty of care to disclose genetic risk information to a patient's relatives on the facts alleged is arguable. This decision could mark the beginning of a legal obligation to disclose patient information to relatives. Indeed, despite the fact that the case concerns a strike out application, Gilbar and Foster argue that it is likely to be regarded not as merely a statement of what the law might arguably be, but what the law is (Gilbar and Foster, 2018).

Genetics professionals often go to significant lengths to ‘persuade’ patients to disclose to relatives and ‘reinforce the professional view that disclosure is important’ (Clarke et al., 2005). However, one study found that whilst a while a significant minority seriously considered informing relatives without consent, only a single geneticist and a single counsellor reported having done so, patient confidentiality and the clinicain's legal liability were the primary reasons for non-disclosure. (Clarke et al., 2005). The mainstreaming of genetic and genomic testing from clinical genetics to other clinical specialties (Annual Report of the Chief Medical Officer, 2017) has raised the profile of the dilemma that clinicians confront when faced with a patient who refuses to pass information on to relatives that the clinician thinks the relative should know. Recent research found that healthcare professionals perceive a moral responsibility rather than a legal responsibility to inform relatives, particularly with treatable conditions (Dheensa et al., 2016 (b)). This work found that a substantial number of nurses working outside genetics felt they should “take steps to inform” relatives if a patient refused to do so in the case of fragile X - 17.8%, breast cancer - 30.1% and Huntington's Disease - 24.7%. However, for each condition, approximately 85% also agreed that they should respect confidentiality. Thus we see that the healthcare professionals continue to find it difficult to action their sense of moral responsibility where they do not perceive the back up of a corresponding legal responsibility and more importantly where they perceive a legal responsibility which requires them not to disclose. There is some evidence that divergence between views of moral and legal responsibilities might not be as wide in the context of clinical genetics where there is growing support for the position that disclosure in the case of actionable genetic conditions should be the default (Parker and Lucassen, 2004). However, the clinician's failure to inform the daughter of her risk in *ABC* suggests that clinicians in other specialties do find it difficult to disclose genetic information against a patient's will even when this conflicts with their professional view of what is right.

In the context of familial disclosure of genetic information, consent provides the only *legal* basis for setting aside the duty of confidence. Professional guidance provides that clinicians have a discretion to disclose but the guidance does not clearly articulate what the *legal* basis is for setting aside the duty of confidence in the exercise of this discretion. The relative's interest in knowing the information does not in and of itself provide a *legal* basis for setting aside the duty of confidence. Thus, the law does not provide support for disclosure in the absence of consent. Despite this, professional guidance *does* recognise that the duty to maintain confidence is not absolute and that ‘it may be justified to breach confidence where the aversion of harm by the disclosure substantially outweighs the patient's claim to confidentiality’ (Royal College of Physicians, Royal College of Pathologists and the British Society of Human Genetics, 2006; GMC, 2017; Nuffield Council on Bioethics, 2015). This lack of legal support for the existing professional discretion makes it difficult to exercise that discretion.

Nevertheless, professionals are instrumental in determining legal standards of care. The *Bolam* test starts with the professional perspective: ‘A doctor is not guilty of negligence if he has acted in accordance with a practice accepted as proper by a responsible body of medical men skilled in that particular art’ ([1957] 1 WLR 582). However, the courts rely on this notion of responsibility to reach a justifiable and fair legal decision, rather than simply allowing the profession to condone any minimally accepted practice. In Bolitho v City and Hackney Health Authority ([1998] AC 232) the House of Lords qualified the *Bolam* standard and asserted the court's ultimate authority in determining expert medical testimony. This confirmed the position that a defendant doctor cannot escape liability merely on the basis of peer's supporting evidence. Lord Browne-Wilkinson said:

The use of these adjectives — responsible, reasonable and respectable — all show that the court has to be satisfied that the exponents of the body of medical opinion relied upon can demonstrate that such opinion has a logical basis ([1998] AC 232).

However, in the context of the informational requirements of a consent to medical treatment, as opposed to the issue of technical medical treatment and diagnosis, the legal standard is no longer referenced to professional opinion. The Supreme Court's decision in Montgomery v Lanarkshire Health Board ([2015] UKSC 11) marked a shift away from reliance on professional expertise in determining disclosure of alternatives and risks in obtaining consent to a medical intervention, to a position which reflects the perspective of the reasonable person in the patient's position. Health professionals are:

under a duty to take reasonable care to ensure that the patient is aware of any material risks involved in any recommended treatment, and of any reasonable alternative or variant treatments. The test of materiality is whether, in the circumstances of the particular case, a reasonable person in the patient's position would be likely to attach significance to the risk, or the doctor is or should reasonably be aware that the particular patient would be likely to attach significance to it ([2015] UKSC 11).

According to the Supreme Court, because these matters were not ‘dependant on medical expertise’ ([2015] UKSC 11), they fell outside the scope of *Bolam.*

Some commentators argue that *Montgomery* concerned the question of whether, as a matter of clinical expertise, to offer a caesarean section as opposed to a vaginal delivery (Montgomery and Montgomery, 2016). They note that in the circumstances, a caesarean would have been outside the guidelines of the Royal College of Obstetricians and Gynaecologists (RCOG) and the National Institute for Health and Care Excellence (NICE) (Montgomery and Montgomery, 2016). This led them to conclude that:

A clinician seeking to avoid legal liability can therefore no longer regard compliance with professional guidelines as a protection but must consider which aspects will be accepted by the judiciary and which not (Montgomery and Montgomery, 2016).

This demonstrates that reliance on professional guidance does not provide guaranteed legal support. The Supreme Court's observation in *Montgomery* that the issue was not ‘dependant on medical expertise’ is also applicable where the concern is whether a patient's genetic information should be passed on to their at-risk relatives. In addition to not ‘depending on medical expertise,’ the practice, and corresponding professional guidance around non-consensual disclosure of genetic information to at-risk relatives is less clear than the guidance addressing appropriate treatment and disclosure of risks as in determining when caesarean section is an appropriate treatment decision (The Royal College of Obstetricians and Gynaecologists, Green-top Guideline No. 42. Shoulder Dystocia. London, second ed., 2012).. In *ABC* both the claimant and the defendant relied on the same professional guidance to support their case. With regard to this professional guidance, the Court of Appeal acknowledged that:

The Human Genetics Commission, the Nuffield Council on Bioethics and the GMC have all expressed the view that the rule of confidentiality is not absolute. In special circumstances it may be justified to break confidence where the aversion of harm by the disclosure substantially outweighs the patient's claim to confidentiality. Examples may include a person declining to inform relatives of a genetic risk of which they may be unaware ([2017] EWCA Civ 336).

Lord Irwin argued that professional opinion would be the first step in determining how to balance conflicting legal duties:

if the clinician conducts the requisite balancing exercise, and concludes that it falls in favour of disclosure then a professional obligation arises. The question is whether a breach of that obligation is actionable ([2017] EWCA Civ 336).

In the event of the establishment of a legal duty, he said that the courts would allow ‘considerable latitude to clinicians faced with such a dilemma’ but that this would be ‘qualified by the consideration that the professional decision must be a reasonable one’ ([2017] EWCA Civ 336).

## 4 Balancing interests in disclosure and non-disclosure

Where consent to familial disclosure of genetic information is refused, evidence demonstrates that this largely prevents disclosure (Clarke et al., 2005; Dheensa et al., 2016 (b)). The public interest exception does not provide a justification for breach of confidence because a relative might benefit. (Mitchell et al. 2017). In English common law, the cases where the public interest in disclosure has been held to outweigh the public interest in maintaining confidence have generally concerned wider public safety (W v Egdell [1990] Ch 359), or issues of public health (Lewis v Secretary of State for Health [2008] EWHC 2196). Indeed, in *ABC* the High Court felt that the public interest in disclosure could not outweigh the public interest in preserving confidence because; ‘what was put against the public interest in preserving confidence … was not a public interest in disclosure, but the private interest of the claimant’ ([2015] EWHC 1394 (QB)). Despite this, the position in professional guidance is that ‘there can be a public interest in disclosing information if the benefits to an individual or society outweigh both the public and the patient's interest in keeping the information confidential’ (GMC, 2017).

The position that the duty to maintain confidentiality should not be absolute in the context of familial genetic medicine is acknowledged in the 2017 Chief Medical Officer's Report (Annual Report of the Chief Medical Officer, 2017), which recognises that ‘genomics offers benefits and responsibilities for the individual, (and) the family …. .that cannot be realised by keeping the secrets revealed from one genome separate from others. The Report continues we need: ‘new ways of thinking about …. confidentiality and caring for families,’ suggesting that: ‘one way forward is for the boundaries of confidentiality in genomics to be seen, at least in some situations, at a familial rather than individual level’. Professional documents therefore recognise that the relative's interest in disclosure can outweigh the patient's interest in non-disclosure. Recognising this professional discretion in *ABC,* Lord Irwin felt that in a situation where professional judgment falls in favour of disclosure, it was ‘not necessarily correct that the law should so clearly incentivise obligations in one direction but not the other’ ([2017] EWCA Civ 336).

Currently the legal incentive to maintain confidence prioritses non-disclosure and inhibits the exercise of professional discretion. The co-existence of legal duties would provide legal protection where the professional balancing exercise concludes in favour of disclosure as well as in those cases where the professional balancing exercise concludes in favour of non-disclosure. However, where these interests are of equal legal weight, it is not clear how a particular interest would achieve priority. Although the professional balancing exercise will be a crucial consideration, we also know the courts will retain their ultimate arbiter role. In *ABC* Lord Irwin indicated some of the features that might inform a court's decision that the duty to disclose emerges as the priority for legal protection. In particular, he felt that protecting the relative's interest in disclosure might be prioritised where the relative ‘should become a patient’ or would ‘require treatment, potentially life-saving in its effect’ ([2017] EWCA Civ 336). However, in *ABC* the focus was on whether there had been a negligent failure to disclose as opposed to whether there had been a breach of confidence. Thus, the judges were concerned with the features of the situation that might tip the balance in favour of disclosure and there was no corresponding discussion of the features of the patient's situation that might be taken into account in determining the weight to be accorded to their interest in confidentiality. Where the clinician is weighing the interests in the balance prior to a making a decision regarding disclosure, there should also be an assessment of the other side of the coin: that is, alongside the features of the situation which weigh in favour of the relative's interest in disclosure, what features of the situation should be considered in valuing the patient's interest in maintaining confidence. Thus, as well as determining what the relative stands to gain from disclosure of the patient's confidential medical information, a thorough and defensible balancing process needs to consider what the patient stands to lose if confidence is breached (Birkhäuer et al., 2017).

## 5 Protecting privacy through breach of confidence

In recent decades, English law on confidentiality has developed significantly outside the context of the clinician-patient relationship (OBJ Ltd v Allan [2008] 1 AC 1; Campbell v Mirror Group Newspapers [2004] UKHL 22; Murray v Express Newspapers [2008] EWCA Civ 446; Douglas v Hello (No. 1) [2001] QB 967). This development has been heavily influenced by the need to give effect to Article 8 of the European Convention on Human Rights which protects people against infringements of privacy. Here the central issue in determining the weight of the confidant's interest in keeping the information confidential has been the reasonableness of his or her expectation that the information will be kept *private* as opposed to the issue of whether the confidant has, or would have refused to consent disclosure (Campbell v Mirror Group Newspapers [2004] UKHL 22; R (on the application of W, X, Y and Z) v Secretary of State for Health [2015] EWCA Civ 1034). In *Campbell v MGN* Lord Hope said:

… a duty of confidence will arise whenever the party subject to the duty is in a situation where he knows or ought to know that the other person can reasonably expect his privacy to be protected ([2004] UKHL 22).

On this basis, the duty of confidence protects privacy where protection of privacy can be reasonably expected. To attract a duty of confidence the law traditionally required the information to ‘have the necessary quality of confidence’ One of the elements of this quality was that the information must have been communicated in circumstances importing an obligation of confidence (Coco v A N Clark (Engineers) Limited [1969] RPC 41). The clinician – patient relationship is a typical example of circumstances which import an obligation of confidence. This obligation enabled the law to protect abuse of trust via its protection of private information. However, English law on breach of confidence has evolved so that it is no longer based on an abuse of trust (Phillipson, 2003). In *Campbell* Lord Nicholls said:

A breach of confidence was restrained to a form of unconscionable conduct, akin to a breach of trust. Today this nomenclature in misleading …. .This cause of action has now firmly shaken off the limiting constraint of the need for an initial confidential relationship. In doing so it has changed its nature …. The more natural description today is that such information is private. The essence of the tort is better encapsulated now as misuse of private information ([2004] UKHL 22).

Rather than the question of whether there was a confidential relationship, Lord Hope felt that:

The underlying question in *all cases* where it is alleged that there has been a breach of the duty of confidence is whether the information disclosed was private … There must be some interest of a private nature that the claimant wishes to protect ([2004] UKHL 22).

The Nuffield Council supports this position and states that: ‘confidentiality is one … of the tools used to achieve and maintain privacy’ (Nuffield Council on Bioethics, 2015). Similarly, the National Data Guardian states:

The common law duty of confidence entitles a patient who consults a doctor to have a reasonable expectation of privacy and this requires the doctor to maintain confidentiality …. (https://assets.publishing.service.gov.uk/government/uploads/system/uploads/attachment_data/file/663089/Exploring_consensus_on_reasonable_expectations_-_July_2017_seminar_FINAL.pdf).

Thus, generally speaking, the emphasis in the action for breach of confidence has shifted from the relationship between the parties and the concept of consent, to the nature of the information and the expectations of the confidant. Of course, clinicians and their patients *do* have relationships and it is these relationships that enable consent. However, if relationships are not crucial to the existence of a duty of confidence, on the other side of the coin, it might be argued that confidence should not be maintained on the basis of the existence of that relationship alone. If relationship alone *is* sufficient to require a healthcare professional to maintain confidence without taking into consideration any of the other circumstances in determining whether information should or should not be disclosed, there is no scope for the protection of other interests because it is axiomatic that relationship will always be present in healthcare professional-patient interactions. The implication of the Court of Appeal's decision in *ABC* is that this relationship, in and of itself, is *not* sufficient to require the clinician to maintain confidence, thereby according with the more recent approach in the common law which, on the other side of the coin, does not require such a relationship for the duty to crystallise. In line with *Campbell* the indication is that it is the nature of the information that is the key legal consideration.

## 6 Determining reasonable expectations of privacy in the context of familial disclosure of genetic information

Managing the familial disclosure of genetic risk information on the basis of patient consent affords protection to the subjective choice of individuals. Managing disclosure on the basis of ‘reasonable expectations of privacy’ presents an objective approach which would allow like cases to be treated alike and could prevent unreasonable non-disclosures. In creating this objective approach, the perspective from which reasonable expectations are determined needs to be established.

In *Campbell* Baroness Hale confirmed that the perspective for assessing the ‘reasonable expectation of privacy’ is that of the subject of the information:

‘reasonable expectations’ are determined by reference to ‘the sensibilities of a reasonable person placed in the situation of the subject of the disclosure’ ([2004] UKHL 22).

On this basis, if a patient of ordinary sensibilities could be said to have a ‘reasonable expectation of privacy’ a disclosure could constitute an infringement of privacy and give rise to a cause of action for breach of confidence (Chico and Taylor, 2018). Where consent and the subjective choice it protects, is not the lawful basis for setting aside the duty of confidence a patient-focused perspective for determining when it is reasonable to expect that information should not be disclosed to relatives on the basis of privacy maintains some protection for the interests of the information subject.

On the face of it, patients have a ‘reasonable expectation of privacy’ whenever they disclose information to a clinician in the clinical context. However, this expectation is based on the existence of the clinician-patient relationship which, as argued above, should not provide the sole criterion for establishing a duty of confidence.

Consent to disclosure would continue negate a breach of confidence. The cases where the concept of ‘reasonable expectation of privacy’ developed did not concern situations where there was an explicit objection to disclosure as there was in *ABC.* However, it was, to varying degrees, clear in those cases that, if given an opportunity to object to the disclosure, the subject of the information would have objected (Campbell v Mirror Group Newspapers [2004] UKHL 22; R (on the application of W, X, Y, and Z) v Secretary of State for Health (British Medical Association intervening) [2015] EWCA Civ 1034). However, it is not clear that an objection would make an otherwise unreasonable expectation of privacy reasonable. Previously courts in the UK have indicated that although the absence of consent might be a factor in determining whether a person could be said to have a ‘reasonable expectation of privacy’, this would not be determinative (JR 38 [2015] UKSC 42; Murray v MGN [2007] EWHC 1908 (Ch)).

Consider the situation in *W, X, Y and Z* ([2015] EWHC 1034), here patient information was passed by NHS Trusts to the Secretary of State for Health and then to the Home Office for the purposes of imposing immigration sanctions applicable in case of certain debts being owed to the NHS. The Court of Appeal held that the patients could ‘not have a reasonable expectation of privacy in the information so far as the Secretary of State and the Home Office are concerned’ ([2015] EWHC 1034). The Court emphasised that the claimants had been made aware of the fact that information about charges incurred would be passed to the Secretary of State and the Home Office. However, the claimant did not give a clear consent to, or refusal of, this use. They consented to treatment, but it is far from clear that consent to this use was entailed in the consent to treatment. Would a refusal have made it reasonable for the patients to expect privacy in the circumstances? Where the objective position is that there cannot be a ‘reasonable expectation of privacy’, as in *W, X, Y and Z*, a refusal to share prima facie reflects an unreasonable expectation of privacy. It follows that this may not require legal protection if the legal test is whether the expectation of privacy is reasonable.

Although *Campbell* determined that the perspective for the ‘reasonable expectation of privacy’ is that of the subject of the information ([2004] UKHL 22), there was little discussion of how this perspective would be informed. In the context of disclosure of patient health data, there is a growing body of research investigating patient and public attitudes to privacy in the context of the use of their personal information that could inform this objective patient-focused position.

The concept of reasonable expectations is also at the heart of the NDG's work which considers the limits of implied consent in negating a breach of confidence in the context of use of patient data (https://www.gov.uk/government/uploads/system/uploads/attachment_data/file/668727/830_-_Supporting_health_and_care_professionals_to_share_data_in_line_with_patient_expectations_-_October_2017_seminar_FINAL.pdf). In addition, in the specific context of familial sharing of genetic information, there is a significant body of empirical work documenting patient and public attitudes to familial sharing. This work demonstrates that most patients do not resist passing on genetic information to their relatives when it would be of benefit (Wiens et al., 2013). Indeed, evidence demonstrates that cases of non-disclosure represent less than 1% of genetic clinic consultations (Clarke at al., 2005). Strikingly, one study found that the ‘majority of individuals believe that affected individuals are *obligated* to disclose genetic information to family members’ (Vavolizza et al., 2015). A study estimating the views of the British public found that in the case of a fatal and preventable disease 93% of the British public would be willing to forgo their confidentiality, if their genetic information could benefit their relatives, with 72% of people feeling strongly that they would be willing to forgo confidence (Heaton and Chico, 2016). Vavolizza et al. confirmed this position with one participant summing the position up thus:

You should never keep medical/health history out of your family. [They] have a right to know (Vavolizza et al., 2015).

Dheensa reports that an overwhelming majority of patients think that their genetic information should not be withheld from relatives on the basis that it is private:

none [of the participants] thought that HCPs should respect patients' refusals on the basis that the information was private and personal to them (Dheensa et al., 2016 (a)).

Indeed, she found that many patients assume that a familial approach is happening and are surprised to hear that sharing and familial use of genetic information are not standard practice.

This overwhelming support for sharing supports the position that most people would not reasonably expect their privacy to be maintained by withholding information from their relatives. In the genetic medicine context one very recent study found that some participants expressed the view that asking patients' permission was undesirable as it would give them an opportunity to refuse. This evidence indicates that there is an objective position which holds that where relatives have the opportunity to access medical treatment upon knowing genetic information, the patient does not have a reasonable expectation of privacy in that information which they can rely upon to prevent disclosure to an at-risk relative. However, this objective picture should not be the end of the assessment of whether the patient's expectations of privacy are reasonable. There may be particular features of a situation which indicate that the patient has compelling reasons for expecting their privacy to be protected, such that their expectation of privacy *is* deemed to be reasonable.

An approach which enquires into the reasons behind a person's decision would be unusual in English medical law, which does not typically enquire into the reasonableness of individual choices. Indeed, it respects choice:

notwithstanding that the reasons for making the choice are rational, irrational, unknown or even non-existent (Re T (Adult: Refusal of Medical Treatment) [1993] Fam. 95).

However, if reasonableness *is* a feature of a legally protectable interest in privacy, the reasoning of those who object becomes relevant in determining the reasonableness of their expectation that their privacy be so protected. As a starting point here it might be argued that a non-disclosure which is protectable on the basis that it reflects a ‘reasonable expectation of privacy’ should be underpinned by reasons that concern protection of the patient's privacy. This might appear trite, but on examination of the extensive empirical literature investigating people's reasons for refusing to disclose genetic risk information to their relatives, we see that patient's reasons for non-disclosure often do not reflect concerns about the impact on their privacy.

There may be many reasons why people want to control the flow of information about themselves. An approach which draws the protection of privacy as arising from the principle of personal autonomy may determine that a desire to control the flow of information about oneself can always be supported by recourse to the principle of privacy. However, the principle of autonomy can be questioned where it is relied on to dominate, control (MacKinnon, 1989), or inappropriately protect others. This raises the question of whether an interest in privacy, which is based on protection of autonomy, should similarly be limited where it is used to dominate, control or inappropriately protect. Rather than allowing people to have recourse to the interest in privacy to control the flow of information about themselves, for any reason, it might be argued that control of information on the basis of the infringement of the interest in privacy should be subject to a narrower interpretation. In *Campbell v MGN* the interest in privacy was held to protect the interest in controlling the dissemination of information about one's private life to retain the right to the esteem and respect of other people ([2004] UKHL 22). Where the patient's concern is not related to the personal impact on the respect and esteem they wish others to hold them in, but reflects a desire to protect or control relatives, the reasonableness of their expectation of privacy, and the corresponding ability to rely on a legal right to privacy to protect that expectation, is questionable.

Evidence suggests that in many cases where a patient refuses to share genetic information with at-risk relatives, their reasons for doing so do not relate to concerns about their privacy in terms of the personal impact they will experience if relatives know this information about them. Rather they are influenced by one of a multitude of other reasons, One study investigating patient's reasons for refusing to disclose genetic information to family members found that in 40 000 clinic based genetic consultations there were 65 cases of non-disclosure, but crucially, protecting privacy was only cited in six cases as the reason for a refusal to disclose (Clarke et al., 2005) Instead, the desire to avoid causing anxiety to relatives was the most frequently cited reason for the refusal to disclose information, with many people worrying about whether their relative(s) could cope with the information. Other common reasons were problematic family dynamics, including loss of contact, the fear of being blamed, an unwillingness to shoulder the responsibility of informing relatives and a general feeling that it was better for the relative not to know about the genetic risk. Several studies support this evidence and also cite the patient's lack of understanding of the genetic condition as a reason for non-disclosure (Vavolizza et al., 2015 Dheensa et al., 2016 (a); Akpinar and Ersoy, 2014; Forrest et al., 2003; Henneman et al., 2002; Gallo et al., 2009). Other empirical work reflects the same concerns but also reports that patients sometimes fail to disclose where they are concerned that their relative will make a choice based on the information that they don't agree with (Dheensa et al., 2017; Henneman L et al., 2002; Gallo et al., 2009).

### 6.1 Practical reasons for decisions not to disclose

Where the reasons for non-disclosure are practical, in that they are based on the patient's distance from, loss of contact with, or dislike for the relative, or the patient's inability to understand and explain the information, it might be argued that an expectation of privacy is not objectively reasonable, and the patient's interest in not disclosing for these practical reasons should not attract legal protection on the basis that he or she has a ‘reasonable expectation of privacy’. Here adequate supporting infrastructure for disclosure and legal protection of health professionals who conduct a careful balancing exercise which concludes in favour of disclosure, are of greater priority than misplaced protection of privacy. The current focus on the importance and potential of genomic medicine in the Chief Medical Officer's Report, and the Court of Appeal's decision in *ABC* that it is arguable that there is a duty of care to disclose genetic information to patients' relatives, ought to provide the impetus for greater infrastructural support for professionals and patients, where these kind of practical concerns prevent the sharing of genetic information which would benefit the patients' relatives.

With adequate resources, clinical genetics services are well placed to fulfil disclosure responsibilities. It is commonplace for practitioners in clinical genetics services go to significant lengths to offer active facilitation of disclosure (Clarke et al., 2005). Familial communication is at the heart of genetic medicine, forming part of the core responsibilities of genetics professionals. Indeed, the Association of Genetic Nurses and Counsellors describes the aims of genetic counselling as ‘to help the individual or family understand the information about the genetic condition, appreciate the inheritance pattern and risk of recurrence, understand the options available and make decisions appropriate to their personal and family situation’ (http://www.agnc.org.uk/media/689675/careerasageneticcounsellor2.pdf). Thus, a patient who wants to withhold information, is likely to present more difficulty for clinical genetics services than a patient who needs practical support to disclose the information.

### 6.2 Protecting relatives as the basis for decisions not to disclose

Reluctance to cause anxiety to relatives is commonly reported to be one of the reasons for non-disclosure of a genetic risk (Forrest.et al., 2003; Featherstone et al., 2006; Vavolizza et al., 2015). The interest that the patient wants to protect in these circumstances does not seem to concern damage to their respect and self-esteem that may occur if others know the particular information about them. This is not to underestimate how difficult it might be for a patient to be the bearer of bad news. However, it does not necessarily follow that people should have a legal right not to disclose, based on protection of their privacy, because they will find disclosure difficult.

In the context of crucial actionable information, which if withheld prevents the relative from becoming a patient, when he or she should become one ([2017] EWCA Civ 336), this protective attitude is misplaced. This kind of protective approach is widely acknowledged to be inappropriate in clinicians' disclosure responsibilities to their patients. Whilst historically clinicians might have sometimes been able to rely on the concept of ‘therapeutic privilege’ to withhold information from their patients, on the basis of the patient's best interests, this paternalistic approach is currently reserved only for very marginal use. The GMC advises:

You should not withhold information necessary for making decisions for any other reason, including where a relative, partner, friend or carer asks you to, unless you believe that giving it could cause the patient serious harm. In this context ‘serious harm’ means more than the patient might become upset or decide to refuse treatment (GMC, 2008).

This privilege does not allow the clinician not to disclose information because he or she judges that a disclosure might cause the patient severe distress or anxiety (Jackson, 2010). A similar paternalistic approach should also not provide the basis for non-disclosure of familial genetic information where the result of a non-disclosure is to deny a capacitated adult vital health choices. In *ABC* the evidence in the High Court demonstrated that this protective desire was a core part of the father's reasons for refusing to allow the clinicians to disclose the information about the risk of Huntington's disease to his daughter. The notes record that the father felt his daughters should not be informed because ‘they might get upset, kill themselves, or have an abortion.’ They also record the professional's concern as to the wisdom of this decision ([2015] EWHC 1394), thereby raising questions about the reasonableness of an approach which *protects* a relative from knowing information, which leads to an inability to *protect* herself from adverse health outcomes. Indeed, the daughter's reaction when she was informed of her risk, when the ability to make the crucial health choice had passed, has presumably caused her more anxiety than a timely disclosure would have.

### 6.3 Preventing choice as the basis for decisions not to disclose

In *ABC* the desire to control his daughter's choices by preventing her from having the opportunity to choose was at the heart of the father's reasons for withholding information from his daughter. The Court of Appeal judgment reports that the social worker recorded: ‘he does not want his daughters to know about it, especially the pregnant one, until she gives birth some time in 2010’ ([2017] EWCA Civ 336). He did not say that he did not want her to know at all, but rather that he did not want her to know ‘until she gives birth’. Coupled with his other reasons for not wanting to disclose to his daughter, it seems that he was not concerned about his privacy in the sense of the impact of others' knowledge of the information about him on his sense of respect and self-esteem, but to prevent his daughter from making a choice that he did not support^2^ The refusal to disclose in order to prevent relatives from accessing termination has been reported in other work (Dheensa et al., 2017; Henneman et al., 2002; Gallo et al., 2009).

### 6.4 Privacy as the basis for decisions not to disclose

The assumption which follows from this discussion may be that wherever the patient *is* concerned to protect her privacy, she *does* have a ‘reasonable expectation of privacy’. However, in considering whether the disclosure of the information was in breach of the claimant's common law right to confidentiality, in *W, X, Y and Z* the Court of Appeal held that:

The transmission of the Information infringes a patient's right to privacy only if (i) he or she has a reasonable expectation of privacy in the Information and (ii) the balancing exercise comes down against disclosure. A breach of the fundamental privacy right will not be established unless both (i) and (ii) are satisfied ([2015] EWCA Civ 1034).

Thus, determining whether the patient's privacy ought to be respected is a two stage process which begins with an assessment of whether it is reasonable to expect privacy in the circumstances. Baroness Hale stipulated this two-stage test in *Campbell*:

the ‘reasonable expectation of privacy’ is a threshold test which brings the balancing exercise into play. It is not the end of the story. Once the information is identified as ‘private’ in this way, the court must balance the claimant's interest in keeping the information private against the countervailing interest of the recipient in publishing it. Very often, it can be expected that the countervailing rights of the recipient will prevail ([2004] UKHL 22).

Where the patient *is* in fact trying to protect his or her privacy, the concerns addressed here where the confidant is concerned about factors other than her privacy do not serve to cast doubt on the reasonableness of expectations of privacy. Thus, where the expectation of privacy is objectively reasonable, because most people would expect privacy in the circumstances, and subjectively reasonable, because the subject of the information does not want other people to have knowledge of that fact about her, we move to the second balancing-exercise stage. It is here that the professional balancing exercise will be particularly difficult. However, where that professional balancing exercise is carefully conducted, we know that it would bear great weight in any subsequent legal investigation of how the competing duties should have been weighed.

Although it is not generally necessary to demonstrate harm in an action for breach of confidence, where this interest has to be balanced against competing interests, some clear and tangible harm to the confidant may tip the balance in favour of non-disclosure. Nevertheless, if a ‘reasonable expectation of privacy’ is the basis for protecting a patient's interest in non-disclosure, as opposed to their consent, this difficult balancing exercise will only arise where the patient is seeking to protect his or her *privacy* interest through non-disclosure. Given the above evidence that in 40 000 genetic clinic consultations there were only 65 refusals to disclose, and only 6 of these were based on privacy, we know that the particularly difficult balancing exercise which will require clinicians to judge whether to protect patient privacy, or the relative's interest in avoiding harm, will arise in less than one percent of cases.

## 7 Conclusion

The appropriateness of consent in providing a legal basis for uses of confidential patient information has recently come into question. However, in the context of sharing genetic information in families the consent model endures. This article argues for a move away from the consent model in sharing familial genetic information, to the concept of privacy which is evolving to provide a comprehensive basis for setting aside the duty of confidence where the subject of the information cannot be said to have a ‘reasonable expectation of privacy’.

The significant empirical evidence reflecting patients' overwhelming desires to share information about genetic risks with their at-risk relatives provides the basis for an objective position regarding what it is reasonable to expect in terms of protection of one's privacy in the face of genetic risk information which might enable relatives to avoid harm. This supports an argument that most people would not expect their privacy to be protected in these circumstances, demonstrating that any expectation that privacy will be protected is, on the face of it, *unreasonable*. Where there is an explicit objection to sharing, such that sharing would amount to a breach of confidence, the subjective circumstances of the objection should be considered in an assessment of its reasonableness.

Where the patient's reasons for sharing are not, in fact, to protect their privacy, but instead to control or protect their relatives, the argument that they have a ‘reasonable expectation of privacy’, that ought to be protected by the duty of confidence, can be doubted. Where there cannot be said to be a ‘reasonable expectation of privacy’ the healthcare professional may determine that the need to disclose the information to an at-risk relative overrides the patient's interest in maintaining confidence. If the healthcare professional has a legal duty to disclose where there cannot be said to be a ‘reasonable expectation of privacy’, this should provide a legal basis for setting aside the legal duty to maintain confidence. If there is no breach of the patient's confidence, the clinician can respect the relative's (legal) interest in disclosure without the fear of litigation.^3^ Rather than create anxiety for clinicians, this should enable them to act in line with their professional view in favour of disclosure. Of course, where the professional opinion is that the patient's expectation of privacy is reasonable in the circumstances, the duty to maintain confidence would continue to provide a legal basis for setting aside any duty to disclose.

## Footnotes

2 The choice of termination perhaps represents a particularly emotive issue that might be made upon receipt of genetic information, but disclosure of genetic risk information could lead to other choices which might be the subject of disagreement in families. Perhaps choices to undergo treatments which are experimental, high risk or innovative. Or undergo treatments which are ethically controversial. Or perhaps disagreement could arise from non-medical choices where a relative chooses to live with little regard for their future after discovering information about a genetic risk.

3 This is not to suggest that it will then be easy for clinicians to ignore a patient's refusal. However, if the patient does not have a legal right to refuse to share the information with their relatives, it might make it easier to have conversations about the need to disclose. Indeed, in the context of organ donation, where there is no legal right for relatives to frustrate a deceased person's living decision to donate, the Human Tissue Authority Code of Practice April 2017 acknowledges the difficult position a clinician might be in, if faced with a relative who does not want the deceased's organs to be used in contravention of the deceased's consent to their use. However, the Code advises clinicians that they should encourage the relatives to accept the deceased person's wishes and should make clear that the relatives do not have the legal right to revoke valid consent, see paragraph 37 Human Tissue Authority Code of Practice April 2017.
